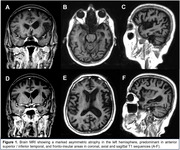# Sporadic mixed primary progressive aphasia with semantic / non‐fluent variant phenotypes and GRN mutation: a case report

**DOI:** 10.1002/alz70857_101997

**Published:** 2025-12-24

**Authors:** Paulo Eduardo Lahoz Fernandez, Paulo Henrique Ferreira Bertolucci

**Affiliations:** ^1^ Federal University of São Paulo ‐ UNIFESP, São Paulo, SP, Brazil

## Abstract

**Background:**

GRN (progranulin) mutation is a common cause of autosomal dominant frontotemporal lobar degeneration (FTLD) but can also occur in non‐familial cases. These mutations usually occur in the behavioral variant FTD (bvFTD) and more rarely in the primary progressive aphasia (PPA), particularly in the non‐fluent / agrammatic (nfvPPA). Recently, it was also described in the semantic (sPPA), logopenic (lvPPA), and mixed (mPPA) variants, suggesting that GRN may cause an overlapping PPA syndrome with a heterogeneous phenotype.

**Method:**

We report the clinical, genetic, and imaging features of a rare case of a patient with Sporadic primary progressive aphasia with mixed phenotype and GRN mutation.

**Result:**

A right‐handed 68‐year‐old woman with 9 years of progressive course of language impairment, showing difficulties in naming objects, comprehending words, and talking over the telephone. The spontaneous speech output was slow and labored, with poor syntax, limited to few words or simple phrases, and repetition possible only for simple sounds. This communication problem led her to avoid conversations, impacting her daily activities. She was a primary education teacher, her medical family history was unremarkable, and the neurological exam was normal. On neuropsychological evaluation, she presented a mixed phenotype (nfvPPA and sPPA), showing anomia, single‐word comprehension/object knowledge, apraxia of speech, and agrammatism. She scored 15/30 on MMSE, 3 on semantic verbal fluency, and 10/20 on the Boston Naming Test. Brain MRI showed a marked asymmetric atrophy in the left hemisphere, predominant in anterior superior / inferior temporal, and fronto‐insular areas in coronal, axial and sagittal T1 sequences. The genetic testing confirmed the GRN gene mutation.

**Conclusion:**

The mixed PPA‐GRN can show multiple levels of language impairment simultaneously altered, such as grammatical, verbal fluency, and semantic, affecting different language pathways, as in this case. The high prevalence of patients with PPA without a family history of FTLD indicates that genetic studies should not be limited to familial cases. Also, GRN mutations are usually present on atypical/mixed PPA phenotypes, suggesting that GRN should be considered primarily in patients with this clinical pattern.